# Habitual dietary intake of flavonoids and all-cause and cause-specific mortality: Golestan cohort study

**DOI:** 10.1186/s12937-020-00627-8

**Published:** 2020-09-28

**Authors:** Jalal Hejazi, Matin Ghanavati, Ehsan Hejazi, Hossein Poustchi, Sadaf G. Sepanlou, Masoud Khoshnia, Abdolsamad Gharavi, Amir Ali Sohrabpour, Masoud Sotoudeh, Sanford M. Dawsey, Paolo Boffetta, Christian C. Abnet, Farin Kamangar, Arash Etemadi, Akram Pourshams, Akbar FazeltabarMalekshah, Paul Brennan, Reza Malekzadeh, Azita Hekmatdoost

**Affiliations:** 1 Department of Nutrition, School of Medicine, Zanjan University of Medical Sciences, Zanjan, Iran; 2 Digestive Oncology Research Center, Digestive Diseases Research Institute, Shariati Hospital, Tehran University of Medical Sciences, Tehran, Iran; 3 Departments of Clinical Nutrition and Dietetics, Faculty of Nutrition and Food Technology, National Nutrition and Food Technology Research Institute, Shahid Beheshti University of Medical Sciences, Tehran, Iran; 4 Liver and Pancreaticobiliary Disease Research Center, Digestive Diseases Research Institute, Shariati Hospital, Tehran University of Medical Sciences, Tehran, Iran; 5 Golestan Research Center of Gastroenterology and Hepatology (GRCGH), Golestan University of Medical Sciences, Gorgan, Iran; 6 Digestive Disease Research Center, Digestive Research Institute, Shariati Hospital, Tehran University of Medical Sciences, Tehran, Iran; 7 Metabolic Epidemiology Branch, Division of Cancer Epidemiology and Genetics, National Cancer Institute, Bethesda, MD USA; 8 Icahn School of Medicine at Mount Sinai, New York, NY USA; 9 Department of Biology, School of Computer, Mathematical, and Natural Sciences, Morgan State University, Baltimore, MD USA; 10 Genetic Epidemiology Group, International Agency for Research on Cancer (IARC / WHO), Lyon, France

**Keywords:** Flavonoids, Mortality, Cardiovascular diseases, Cancer

## Abstract

**Background and objectives:**

Flavonoids are the most important group of polyphenols with well-known beneficial effects on health. However; the association of intake of total flavonoid or their subclasses with all-cause or cause-specific mortality is not fully understood. The present study aims to evaluate the association between intake of total flavonoid, flavonoid subclasses, and total and cause-specific mortality in a developing country.

**Methods:**

A total number of 49,173 participants from the Golestan cohort study, who completed a validated food frequency questionnaire at recruitment, were followed from 2004 till 2018. Phenol-Explorer database was applied to estimate dietary intakes of total flavonoid and different flavonoid subclasses. Associations were examined using adjusted Cox proportional hazards models.

**Results:**

During a mean follow-up of 10.63 years, 5104 deaths were reported. After adjusting for several potential confounders, the hazard ratios (HRs) of all-cause mortality for the highest versus the lowest quintile of dietary flavanones, flavones, isoflavonoids, and dihydrochalcones were 0.81 (95% confidence interval = 0.73–0.89), 0.83(0.76–0.92), 0.88(0.80–0.96) and 0.83(0.77–0.90), respectively. However, there was no association between total flavonoid intake or other flavonoid subclasses with all-cause mortality. In cause-specific mortality analyses, flavanones and flavones intakes were inversely associated with CVD mortality [HRs: 0.86(0.73–1.00) and 0.85(0.72–1.00)] and isoflavonoids and dihydrochalcones were the only flavonoid subclasses that showed a protective association against cancer mortality [HR: 0.82(0.68–0.98)].

**Conclusion:**

The results of our study suggest that certain subclasses of flavonoids can reduce all-cause mortality and mortality rate from CVD and cancer.

## Introduction

Flavonoids, an important subgroup of polyphenols, have a substantial impact on different aspects of health. They have attracted considerable attention during recent decades due to their abundance in the diet and potential health effects [1]. Over the past two decades, a large number of studies have investigated the effects of various flavonoids, (e.g. flavonols, flavones and isoflavones) or their rich sources (e.g. green tea, dark chocolate, and red wine) on degenerative diseases such as cardiovascular disease and cancer, with results favoring protection against these diseases [2–5].

Dietary flavonoids are chemically diverse and are divided into 6 main subclasses, i.e. flavanols or flavan-3-ols (e.g. catechin, epicatechin, epigallocatechin), anthocyanins (e.g. cyanidin, pelargonidin, delphinidin, peonidin), flavanones (e.g. hesperetin, naringenin), flavonols (e.g. quercetin, kaempferol, myricetin), flavones (e.g. apigenin, luteolin), isoflavones (e.g. daidzein, genistein) and some subsidiary classes such as dihydrochalcones and chalcones (e.g. phloridzin, arbutin, phloretin, and chalconaringenin) [6]. These subclasses vary in their biological efficacy and bioavailability.

Most of the beneficial effects of flavonoids intake are attributed to their antioxidant and anti-inflammatory characteristics; however, some recent studies have suggested several mechanisms for their anti-mutagenic and anti-carcinogenic properties [7]. Some phenolic compounds such as catechins [8], hesperetin [9], and genistein [10] are among the most well-known compounds in this regard.

Although numerous studies have tried to substantiate if there is a viable relationship between consumption of some flavonoids or their food sources and a specific disease or health condition, there are relatively few studies concerning the correlation between total flavonoid or flavonoid subclasses intake and all-cause or cause-specific mortality. The results of a recent Australian cohort study showed that individuals in the highest tertile of intake of total flavonoid and its subclasses had a significantly lower all-cause mortality compared with those in the lowest tertile [11]. However, in another large scale recent cohort study (Nurses’ Health Study II), no significant association was shown between total flavonoid or flavonoid subclasses and all-cause mortality [12]. There are also conflicting results on the associations between the different subclasses of flavonoids intake and all-cause mortality, as well as the association between mortality from specific causes (eg, mortality from CVD or cancer) and total flavonoid or flavonoid subclasses. Just as an example, in a meta-analysis of 14 cohort study, Wang et al. have concluded that dietary intakes of all six flavonoid subclasses are associated with lower risks of CVD [13]; however, in a more recent large scale cohort of Framingham Offspring, the authors have reported only a significant inverse association between higher intake of flavonols and CVD incidence, but not for the other flavonoid subclasses [14].

Because of these inconsistent results, there is a need for several large-scale and well-designed studies to expand our knowledge concerning the role of flavonoids in reducing all-cause and cause-specific mortality risks. Golestan cohort study (GCS), a large-scale prospective study in the northeast of Iran, used a comprehensive and validated food frequency questionnaire and an accurate cause of mortality ascertainment, thus providing an excellent opportunity to assess the relationship between dietary total flavonoid and flavonoid subclasses intake and total and cause-specific mortality.

## Materials and methods

### Population and study design

**Fig. 1:**
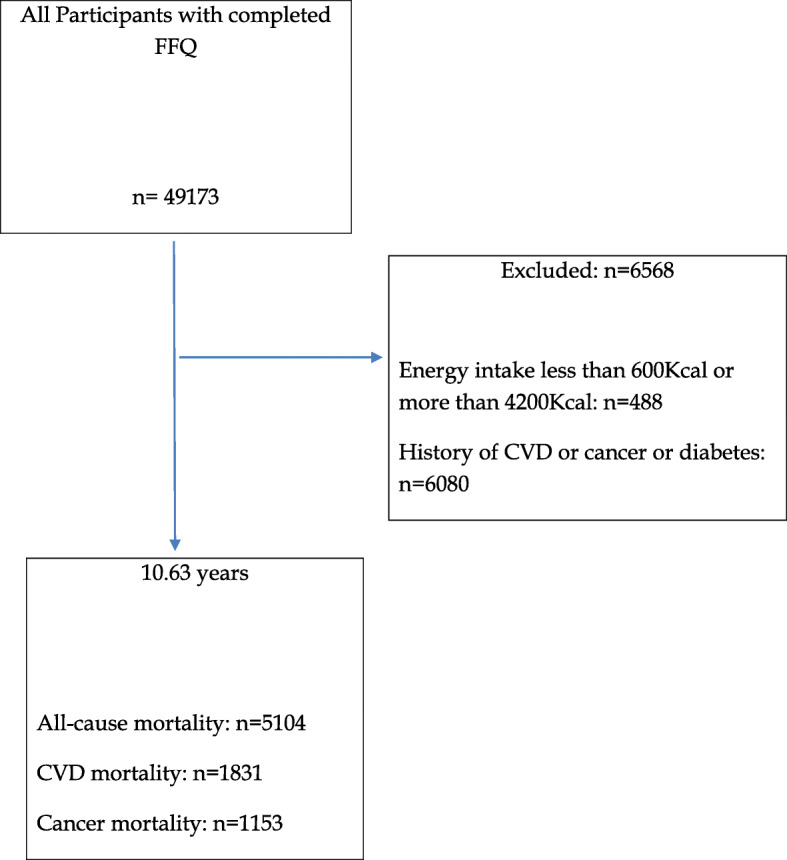
Consort flow diagram. FFQ, food frequency questionnaire, CVD, cardiovascular disease

The study protocol of GCS is explained in detail elsewhere [15]. Briefly, this prospective cohort study was launched and is ongoing in Golestan province in the northeast of Iran. A total number of 50,045 participants were recruited randomly from Gonbad city and 326 surrounding villages and have been followed since 2004. The conduct of GCS was approved by the institutional review boards of the Digestive Disease Research Center of Tehran University of Medical Sciences, the US National Cancer Institute (NCI), and the World Health Organization International Agency for Research on Cancer (IARC). All participants were provided with written informed consent before enrolment. Flow diagram of participants of GCS and those excluded from this analysis is presented in Fig. 1.

### Dietary intake and flavonoids evaluation

Dietary intake was assessed using a 116-item semi-quantitative food frequency questionnaire (FFQ). The validity and reliability of the FFQ have been discussed in detail elsewhere [16]. Information on the usual portion size and the frequency of intake was collected for each food item. Afterward, the reported frequencies and portion sizes for each food item were converted to average daily intake in grams. For calculating energy intake, the consumed amount of each food item (g/day) was multiplied by the energy composition of each item, using the Iranian [17] and US Department of Agriculture [18] databases.

For each food, the mean content in different subclasses of flavonoids was calculated using the Phenol-Explorer database (www.phenol-explorer.eu/contents). With the aid of this information and the daily consumption of each food source, the intakes of seven subclasses of flavonoids (Flavonols, Flavan-3-ol monomers, Flavanones, Flavones, Anthocyanins, Isoflavonoids, and Dihydrochalcones) were calculated for all participants.

### The assessment of potential confounders

At the beginning of the study, trained physicians used a structured questionnaire to collect data on demographic, lifestyle, and medical history information during face-to-face interviews. The participants provided information on their gender, age, literacy (literate vs. illiterate), residence (urban vs. rural), socio-economic status, tobacco smoking, opium use, alcohol consumption, physical activities, and their medical history including self-reported history of cardiovascular diseases (heart disease and/or stroke), hypertension and diabetes.

Physical examination including anthropometric and blood pressure measurements was performed by trained health personnel. Height, weight, waist, and hip circumference were measured with light clothing. Body mass index (BMI) was calculated by dividing weight (kilograms) by the square of height (meters). Systolic (SBP) and diastolic blood pressure (DBP) were measured using Richter auscultatory sphygmomanometers twice in a sitting position on the right arm after a five-minutes rest. The mean of the two measurements was considered as the subject’s blood pressure.

### Ascertainment of the cause of death

All participants in the study were contacted annually through telephone calls. When death was reported, the GCS team visited the participant’s dwelling and the medical centers in which the person’s medical needs were attended. The team collected all clinical reports, pathology reports, hospital records, and any available tumor samples. Additionally, the team completed a validated verbal autopsy questionnaire [19] to determine the possible cause of death. In this study, we considered total deaths in general and deaths due to CVD and cancer (all cancers together and GI cancers) in particular.

### Statistical analysis

The baseline characteristics of all participants compared by quintiles of total flavonoid intake. Descriptive data were presented as mean ± SD for continuous variables and frequencies, and percentages for categorical variables. One-factor ANOVA or Pearson chi-squared tests were used to compare the quantitative or categorical baseline characteristics of the participants across quintiles of baseline total flavonoid intake. Person-time for each participant was calculated from the date of the completion of the questionnaires to the date of death or the last follow-up (August 2018), whichever came first.

Time-dependent Cox proportional hazard ratios (HR) and 95% confidence intervals (CIs) for all-cause, CVD- and cancer-related mortality were computed using quintiles of the exposure variables, where the lowest quintile (reflecting the lowest intakes) was the referent category. Schoenfeld residuals were used to check the Cox proportional hazards assumptions, with no evidence of violation for all outcomes Cox proportional hazard regressions. Known confounders including BMI, education level, physical activity, tobacco smoking, opiate use, age, gender, total energy intake, history of diabetes, and hypertension were controlled by using multivariate models.

The data were analyzed using STATA software, version 12.0 (Stata Corp., College Station, TX, USA). All tests were two-tailed and *p*-values below 0.05 were considered significant.

## Results

A total of 42,605 participants were entered into the analysis. The characteristics of the participants based on quintile of total flavonoid intake are shown in Table 1. The participants with higher flavonoid intake had higher calorie intake but lower BMI compared with those with lower polyphenol intakes. They also had a lower smoking rate but higher alcohol consumption.

**Table 1:** Characteristics of participants according to quintile of total flavonoid intake

	Total Flavonoid Intake Range					
	All	Q1	Q2	Q3	Q4	Q5
n (%)	42,605	8317 (19.5)	8654 (20.3)	8692 (20.4)	8611 (20.2)	8331 (19.6)
Gender						
Women [n (%)]	24,262 (56.9)	5724 (68.8)	5417 (62.6)	4982 (57.3)	4486 (52.1)	3653 (43.8)
Men [n (%)]	18,343 (43.1)	2593 (31.2)	3237 (37.4)	3710 (42.7)	4125 (47.9)	4678 (56.2)
Age (y) [Mean ± SD]^a^	51.55 (8.78)	52.15 (9.19)	51.30 (8.76)	51.23 (8.62)	51.37 (8.60)	51.75 (8.70)
Mortality No (%)						
Total	5104 (12)	1066 (12.8)	971 (11.2)	995 (11.4)	962 (11.2)	1110 (13.3)
Cardiovascular	1831 (4.3)	408 (4.9)	325 (3.8)	357 (4.1)	357 (4.1)	384 (4.6)
Cancer	1153 (2.7)	213 (2.6)	231 (2.7)	229 (2.6)	216 (2.5)	264 (3.2)
Other cause	2120 (5)	445 (5.4)	415 (4.8)	409 (4.7)	389 (4.5)	462 (5.5)
BMI (Kg/m^2^) [Mean ± SD]^a^	26.45 (5.40)	26.72 (5.58)	26.58 (5.40)	26.48 (5.36)	26.32 (5.30)	26.14 (5.35)
Waist- to – Hip [Mean ± SD]^a^	0.94 (0.08)	0.94 (0.08)	0.94 (0.08)	0.94 (0.08)	0.94 (0.07)	0.94 (0.07)
Energy (kcal) [Mean ± SD]^a^	2169.08 (570.22)	1758.99 (438.61)	2007.29 (449.14)	2169.49 (466.56)	2334.77 (504.15)	2574.85 (584.81)
Smoke ever used [n (%)]^a^						
No smoker	35,230 (82.7)	7389 (88.8)	7454 (86.1)	7312 (84.1)	6972 (81)	6103 (73.3)
Smoker	7375 (17.3)	928 (11.2)	1200 (13.9)	1380 (15.9)	1639 (19)	2228 (26.7)
Alcohol ever used [n (%)]^a^						
No	41,185 (96.7)	8150 (98)	8435 (97.5)	8432 (97)	8312 (96.5)	7856 (94.3)
Yes	1420 (3.3)	167 (2)	219 (2.5)	260 (3)	229 (3.5)	475 (5.7)
Opiate ever use [n (%)]^a^						
No	35,523 (83.4)	7345 (88.3)	7513 (86.8)	7386 (85)	7056 (81.9)	6223 (74.7)
Yes	7082 (16.6)	972 (11.7)	1141 (13.2)	1306 (15)	1555 (18.1)	2108 (25.3)
History of Hypertension [n (%)]^a^						
No	35,751 (83.9)	6613 (79.5)	7212 (83.3)	7360 (84.7)	7389 (85.8)	7177 (86.1)
Yes	6854 (16.1)	1704 (20.5)	1442 (16.7)	1332 (15.3)	1222 (14.2)	1154 (13.9)
Education ^[n (%)]a^						
Illiterate	29,561 (69.4)	6333 (76.1)	6186 (71.5)	6005 (69.1)	5842 (67.8)	5195 (62.4)
< 5 year	7392 (17.4)	1200 (14.4)	1401 (16.2)	1578 (18.2)	1537 (17.8)	1676 (20.1)
6–8 year	1916 (4.5)	278 (3.3)	340 (3.9)	363 (4.2)	424 (4.9)	511 (6.1)
High school	2792 (6.6)	391 (4.7)	523 (6)	566 (6.5)	585 (6.8)	727 (8.7)
Academic	944 (2.2)	115 (1.4)	204 (2.4)	180 (2.1)	223 (2.6)	222 (2.7)
Ethnicity^a^						
Torkaman	32,033 (75.2)	6619 (79.6)	6627 (76.6)	6525 (75.1)	6368 (74)	5894 (70.7)
Non torkaman	10,572 (24.8)	1698 (20.4)	2072 (23.4)	2167 (24.9)	2243 (26)	2437 (29.3)
Marital status^a^						
Married	37,677 (88.6)	7085 (85.4)	7608 (88)	7753 (89.4)	7746 (90.1)	7485 (90)
Not married	4854 (11.4)	1216 (14.6)	1033 (12)	921 (10.6)	854 (9.9)	830 (10)
Physical activity^a^						
Sedentary	14,424 (33.9)	2764 (32.6)	2814 (32.8)	2871 (33.4)	3133 (37.7)	3716 (38.9)
Moderate	13,370 (31.5)	2716 (32.8)	2789 (32.1)	2641 (30.8)	2391 (28.8)	2770 (29)
High	14,709 (34.6)	2816 (34.6)	3045 (35.1)	3075 (35.8)	2782 (33.5)	3063 (32.1)

^a^All comparisons between subclasses of flavonoid quintiles and tests for trend were statistically significant at *P* < 0.001 (chi-squared for categorical variables or Kruskal–Wallis rank sum test for continuous variables)

During 10.63 years of the follow-up period, 5104 deaths cases were reported. There were no marked differences in rates of all-cause mortality, cardiovascular mortality, or cancer mortality by quantiles of total flavonoid intake.

Table 2 shows Cox proportional hazard ratio (HR) and 95% CI for total mortality according to quintiles of total flavonoid and their subclasses. After adjusting for potential confounders such as gender, age, ethnicity, education, marital status, smoking, opium use, alcohol consumption, BMI, hypertension, occupational physical activity and energy intake, participants with higher quantiles of flavanones, flavones, isoflavonoids, and dihydrochalcones had significantly lower all-cause mortality risk compared with those at the lowest group of the consumption (HR: 0.81; CI:0.73–0.89, HR: 0.83; CI:0.76–0.92, HR: 0.88; CI: 0.80–0.96, and HR:0.83; CI:0.77–0.90, respectively).

**Table 2:** Association between dietary flavonoids intake and all-cause mortality

Flavonoids	Quintile of Intake					*p* for trend
	1	2	3	4	5	
**Total Flavonoid**						
Mean intake (mg/d)	212.19	431.24	589.64	758.54	1214.75	
No. of deaths/person-year	979/87,933	939/92,285	976/93,291	1053/93,331	1156/91,508	
Age-adjusted HR (95% CI) ^a^	1	0.98 (0.89–1.07)	1.01 (0.92–1.10)	1.04 (0.96–1.14)	1.19 (1.09–1.29)	< 0.001
Multivariate-adjusted HR (95% CI) ^b^	1	0.97 (0.89–1.07)	0.97 (0.89–1.06)	1.00 (0.91–1.09)	1.08 (0.98–1.18)	0.07
**Flavonols**						
Mean intake (mg/d)	29.63	53.51	70.26	89.08	137.21	
No. of deaths/person-year	990/87,718	937/92,170	950/93,921	1078/92,928	1149/91,657	
Age-adjusted HR (95% CI) ^a^	1	0.96 (0.88–1.00)	0.96 (0.88–1.05)	1.04 (0.95–1.13)	1.15 (1.06–1.26)	< 0.001
Multivariate-adjusted HR (95% CI) ^b^	1	0.96 (0.87–1.04)	0.94 (0.91–1.09)	1.00 (0.90–1.07)	1.06 (0.99–1.18)	0.09
**Flavan-3-ol monomers**						
Mean intake (mg/d)	160.18	347.89	486.03	627.77	1021.65	
No. of deaths/person-year	972/87,919	920/92,249	993/93,239	1054/93,522	1165/91,453	
Age-adjusted HR (95% CI) ^a^	1	0.95 (0.87–1.00)	1.04 (0.95–1.13)	1.03 (0.94–1.12)	1.19 (1.09–1.30)	< 0.001
Multivariate-adjusted HR (95% CI) ^b^	1	0.95 (0.87–1.04)	0.99 (0.91–1.09)	0.98 (0.90–1.07)	1.08 (0.99–1.18)	0.05
**Flavanones**						
Mean intake (mg/d)	0.84	3.13	5.85	10.00	27.79	
No. of deaths/person-year	1305/90,182	1061/92,107	955/92,838	919/90,834	863/92,511	
Age-adjusted HR (95% CI) ^a^	1	0.88 (0.81–0.96)	0.82 (0.75–0.89)	0.80 (0.73–0.87)	0.73 (0.67–0.80)	< 0.001
Multivariate-adjusted HR (95% CI) ^b^	1	0.88 (0.81–0.96)	0.83 (0.77–0.91)	0.84 (0.77–0.92)	0.81 (0.73–0.89)	< 0.001
**Flavones**						
Mean intake (mg/d)	1.55	2.75	3.78	5.18	9.94	
No. of deaths/person-year	1272/87,823	1039/92,062	973/93,032	932/93,820	888/91,717	
Age-adjusted HR (95% CI) ^a^	1	0.88 (0.82–0.96)	0.87 (0.80–0.94)	0.82 (0.76–0.89)	0.77 (0.70–0.84)	< 0.001
Multivariate-adjusted HR (95% CI) ^b^	1	0.91 (0.84–0.99)	0.90 (0.82–0.98)	0.85 (0.78–0.94)	0.83 (0.76–0.92)	< 0.001
**Isoflavnoids**						
Mean intake (mg/d)	0.007	0.024	0.053	0.21	1.68	
No. of deaths/person-year	1255/89,134	1114/92,971	1011/93,204	876/92,648	848/90,486	
Age-adjusted HR (95% CI) ^a^	1	0.93 (0.86–1.01)	0.88 (0.82–0.95)	0.80 (0.73–0.87)	0.81 (0.75–0.89)	< 0.001
Multivariate-adjusted HR (95% CI) ^b^	1	0.94 (0.87–1.04)	0.91 (0.84–0.99)	0.85 (0.78–0.93)	0.88 (0.80–0.96)	< 0.001
**Anthocianidins**						
Mean intake (mg/d)	7.72	15.05	20.20	26.11	41.45	
No. of deaths/person-year	1049/87,580	929/92,598	980/93,136	1031/93,326	1124/91,781	
Age-adjusted HR (95% CI) ^a^	1	0.91 (0.83–0.99)	0.96 (0.88–1.05)	0.98 (0.90–1.07)	1.11 (1.02–1.20)	0.003
Multivariate-adjusted HR (95% CI) ^b^	1	0.93 (0.83–0.99)	0.94 (0.86–1.03)	0.94 (0.86–1.03)	1.03 (0.94–1.13)	0.34
**Dihydrochalcones**		?				
Mean intake (mg/d)	0.017		0.19	0.46	1.66	
No. of deaths/person-year	2204/184,266		1026/90,601	1014/93,067	860/90,570	
Age-adjusted HR (95% CI) ^a^	1		0.97 (0.91–1.04)	0.92 (0.80–0.99)	0.78 (0.72–0.84)	< 0.001
Multivariate-adjusted HR (95% CI) ^b^	1		0.97 (0.90–1.04)	0.94 (0.87–1.01)	0.83 (0.77–0.90)	< 0.001

^a^Age-adjusted model was adjusted for age (years)

b Multivariate-adjusted model was adjusted for gender; age (years); ethnicity (Turkmen, others); education (illiterate, ≤5 years, 6–8 years, high school, academic); marital status (married, not married); smoking (user, non-user); opium use (user, non-user); alcohol (user, non-user); BMI (continuous); hypertension (yes, no); occupational physical activity (sedentary, moderate activity, high activity), energy intake (continuous)

Regarding CVD mortality, after adjusting for confounding variables, only the consumption of flavanones and flavones (HR: 0.86; CI: 0.73–1, and HR:0.85; CI:0.72–1, respectively) had a significant protective effect and there was no association between the intake of other flavonoids and CVD mortality risk (Table 3). Also regarding stroke mortality, after adjusting for confounding variables, flavones, and dihydrochalcones were the only subclasses of flavonoids which showed a beneficial effect (HR: 0.74; CI: 0.56–0.98 and HR: 0.71; CI: 0.56–0.91).

**Table 3:** Association between dietary flavonoids intake and CVD mortality

Flavonoids	Quintile of intake					*p* for trend
	1	2	3	4	5	
**Total Flavonoids**						
**Cardiovascular Disease**						
No. of deaths	370	337	351	380	393	
Age-adjusted HR (95% CI) ^a^	1	0.94 (0.81–1.09)	0.97 (0.84–1.12)	1.01 (0.87–1.16)	1.08 (0.94–1.25)	0.14
Multivariate-adjusted HR (95% CI) ^b^	1	0.96 (0.82–1.11)	0.97 (0.84–1.13)	1.02 (0.88–1.18)	1.06 (0.91–1.23)	0.27
**Age**						
< 60 years						
HR (95% CI) ^b^	1	1.05 (0.86–1.31)	0.96 (0.76–1.20)	1.00 (0.80–1.25)	1.22 (0.98–1.51)	0.12
> 60 years						
HR (95% CI) ^b^	1	0.86 (0.70–1.05)	0.91 (0.74–1.11)	0.94 (0.78–1.15)	0.79 (0.63–0.97)	0.12
**BMI**						
< 30						
HR (95% CI) ^b^	1	0.97 (0.82–1.15)	0.95 (0.81–1.13)	0.98 (0.83–1.16)	1.03 (0.87–1.22)	0.69
> 30						
HR (95% CI) ^b^	1	0.84 (0.61–1.15)	0.93 (0.68–1.28)	0.98 (0.72–1.34)	0.86 (0.61–1.21)	0.72
**Coronary Heart Disease**						
No. of deaths	174	145	147	182	187	
Age-adjusted HR (95% CI) ^a^	1	0.85 (0.68–1.06)	0.85 (0.68–1.06)	0.83 (0.83–1.25)	0.88 (0.88–1.33)	0.14
Multivariate-adjusted HR (95% CI) ^b^	1	0.84 (0.67–1.05)	0.84 (0.67–1.05)	0.99 (0.80–1.23)	1.01 (0.83–1.26)	0.44
**Age**						
< 60 years						
HR (95% CI) ^b^	1	0.83 (0.62–1.12)	0.79 (0.59–1.07)	0.67 (0.49–0.91)	0.87 (0.66–1.15)	0.15
> 60 years						
HR (95% CI) ^b^	1	0.71 (0.52–0.99)	0.81 (0.59–1.10)	0.87 (0.64–1.17)	0.70 (0.51–0.98)	0.16
**BMI**						
< 30						
HR (95% CI) ^b^	1	0.80 (0.62–1.04)	0.84 (0.65–1.08)	0.92 (0.72–1.17)	0.95 (0.74–1.21)	0.74
> 30						
HR (95% CI) ^b^	1	0.92 (0.58–1.44)	0.72 (0.44–1.19)	1.07 (0.68–1.68)	0.94 (0.58–1.53)	0.96
**Stroke**						
No. of deaths	132	117	122	120	129	
Age-adjusted HR (95% CI) ^a^	1	0.93 (0.72–1.19)	0.97 (0.76–1.24)	0.91 (0.71–1.16)	1.02 (0.80–1.30)	0.96
Multivariate-adjusted HR (95% CI) ^b^	1	0.98 (0.76–1.27)	1.01 (0.79–1.30)	0.95 (0.74–1.23)	1.05 (0.81–1.36)	0.79
**Age**						
< 60 years						
HR (95% CI) ^b^	1	1.26 (0.84–1.91)	1.18 (0.78–1.80)	1.02 (0.65–1.58)	1.42 (0.93–2.1)	0.29
> 60 years						
HR (95% CI) ^b^	1	0.83 (0.60–1.15)	0.86 (0.62–1.18)	0.86 (0.63–1.18)	0.79 (0.56–1.11)	0.24
**BMI**						
< 30						
HR (95% CI) ^b^		1.05 (0.79–1.40)	1.02 (0.77–1.36)	0.89 (0.66–1.19)	1.02 (0.76–1.37)	0.89
> 30						
HR (95% CI) ^b^		0.73 (0.41–1.27)	0.91 (0.53–1.56)	0.67 (0.67–1.87)	0.58 (0.58–1.77)	0.52
**Flavonols**						
**Cardiovascular Disease**						
No. of deaths	379	338	337	378	399	
Age-adjusted HR (95% CI) ^a^	1	0.91 (0.79–1.06)	0.90 (0.78–1.04)	0.96 (0.83–1.11)	1.06 (0.92–1.22)	0.26
Multivariate-adjusted HR (95% CI) ^b^	1	0.93 (0.80–1.08)	0.91 (0.79–1.06)	0.98 (0.85–1.14)	1.05 (0.91–1.23)	0.34
**Age**						
< 60 years						
HR (95% CI) ^b^	1	1.03 (0.83–1.29)	0.92 (0.74–1.16)	0.99 (79–1.24)	1.22 (0.98–1.52)	0.11
> 60 years						
HR (95% CI) ^b^	1	0.82 (0.67–1.01)	0.85 (0.70–1.04)	0.90 (0.74–1.09)	0.78 (0.63–0.96)	0.08
**BMI**						
< 30						
HR (95% CI) ^b^	1	0.97 (0.82–1.15)	0.87 (0.60–1.01)	0.96 (0.81–1.14)	1.02 (0.86–1.21)	0.80
> 30						
HR (95% CI) ^b^	1	0.75 (0.55–1.04)	1.00 (0.74–1.35)	0.87 (0.63–1.20)	0.88 (0.63–1.24)	0.73
**Coronary Heart Disease**						
No. of deaths	177	142	145	182	189	
Age-adjusted HR (95% CI) ^a^	1	0.81 (0.65–1.01)	0.82 (0.66–1.02)	0.99 (0.80–1.22)	1.07 (0.87–1.31)	0.17
Multivariate-adjusted HR (95% CI) ^b^	1	0.81 (0.65–1.01)	0.80 (0.64–1.00)	0.97 (0.81–1.25)	1.00 (0.81–1.25)	0.46
**Age**						
< 60 years						
HR (95% CI) ^b^	1	0.84 (0.61–1.15)	0.77 (0.56–1.06)	1.00 (0.74–1.35)	1.13 (0.84–1.53)	0.17
> 60 years						
HR (95% CI) ^b^	1	0.76 (0.55–1.04)	0.79 (0.58–1.08)	0.87 (0.64–1.18)	0.73 (0.52–1.02)	0.19
**BMI**						
< 30						
HR (95% CI) ^b^	1	0.82 (0.63–1.05)	0.78 (0.60–1.01)	0.93 (0.73–1.19)	0.94 (0.73–1.20)	0.93
> 30						
HR (95% CI) ^b^	1	0.74 (0.46–1.18)	0.81 (0.51–1.29)	0.93 (0.58–1.47)	0.94 (0.58–1.53)	0.93
**Stroke**						
No. of deaths	133	123	117	116	131	
Age-adjusted HR (95% CI) ^a^	1	0.96 (0.75–1.23)	0.91 (0.71–1.17)	0.85 (0.66–1.09)	1.01 (0.79–1.29)	0.75
Multivariate-adjusted HR (95% CI) ^b^	1	1.02 (0.79–1.30)	0.97 (0.75–1.24)	0.90 (0.70–1.17)	1.07 (0.82–1.38)	0.95
**Age**						
< 60 years						
HR (95% CI) ^b^	1	1.47 (0.98–2.20)	1.02 (0.66–1.58)	1.03 (0.66–1.61)	1.48 (0.97–2.26)	0.35
> 60 years						
HR (95% CI) ^b^	1	0.78 (0.56–1.08)	0.89 (0.65–1.22)	0.79 (0.57–1.08)	0.78 (0.56–1.10)	0.19
**BMI**						
< 30						
HR (95% CI) ^b^	1	1.10 (0.83–1.46)	0.94 (0.70–1.25)	0.86 (0.64–1.16)	1.04 (0.77–1.40)	0.65
> 30						
HR (95% CI) ^b^	1	0.71 (0.40–1.25)	1.05 (0.63–1.75)	0.98 (0.58–1.67)	1.02 (0.58–1.80)	0.64
**Flavan-3-ol monomers**						
**Cardiovascular Disease**						
No. of deaths	371	330	353	384	393	
Age-adjusted HR (95% CI) ^a^	1	0.90 (0.77–1.04)	0.98 (0.84–1.13)	0.99 (0/92–1.14)	1.06 (0.92–1.23)	0.16
Multivariate-adjusted HR (95% CI) ^b^	1	0.92 (0.79–1.07)	0.97 (0.83–1.12)	1.00 (0.86–1.15)	1.04 (0.89–1.21)	0.47
**Age**						
< 60 years						
HR (95% CI) ^b^	1	1.01 (0.81–1.26)	0.92 (0.73–1.15)	1.00 (0.80–1.25)	1.20 (0.97–1.48)	0.11
> 60 years						
HR (95% CI) ^b^	1	0.83 (0.67–1.02)	0.93 (0.76–1.13)	0.92 (0.75–1.12)	0.76 (0.62–0.95)	0.08
**BMI**						
< 30						
HR (95% CI) ^b^	1	0.95 (0.81–1.13)	0.95 (0.80–1.13)	0.97 (0.82–1.14)	1.01 (0.85–1.20)	0.82
> 30						
HR (95% CI) ^b^	1	0.75 (0.54–1.03)	0.92 (0.67–1.25)	0.95 (0.69–1.29)	0.84 (0.60–1.17)	0.71
**Coronary Heart Disease**						
No. of deaths	177	137	153	179	189	
Age-adjusted HR (95% CI) ^a^	1	0.77 (0.62–0.97)	0.88 (0.70–1/09)	0.96 (0.78–1.19)	1.06 (0.86–1.31)	0.16
Multivariate-adjusted HR (95% CI) ^b^	1	0.77 (0.61–0.96)	0.86 (0.69–1.07)	0.94 (0.76–1.16)	0.99 (0.80–1.23)	0.47
**Age**						
< 60 years						
HR (95% CI) ^b^	1	0.89 (0.65–1.22)	0.78 (0.57–1.08)	1.04 (0.77–1.41)	1.18 (0.88–1.58)	0.12
> 60 years						
HR (95% CI) ^b^	1	0.66 (0.46–0.89)	0.87 (0.64–1.17)	0.79 (0.58–1.06)	0.69 (0.49–0.95)	0.11
**BMI**						
< 30						
HR (95% CI) ^b^	1	0.76 (0.59–0.99)	0.87 (0.68–1.12)	0.87 (0.68–1.11)	0.93 (0.73–1.19)	0.99
> 30						
HR (95% CI) ^b^	1	0.74 (0.46–1.17)	0.70 (0.43–1.14)	1.04 (0.67–1.61)	0.91 (0.57–1.47)	0.85
**Stroke**						
No. of deaths	129	119	122	123	127	
Age-adjusted HR (95% CI) ^a^	1	0.94 (0.73–1.21)	1.00 (0.78–1.28)	0.92 (0.72–1.18)	1.00 (0.79–1.29)	0.98
Multivariate-adjusted HR (95% CI) ^b^	1	1.01 (0.78–1.29)	1.01 (0.78–1.30)	0.97 (0.75–1.25)	1.03 (0.80–1.34)	0.91
**Age**						
< 60 years						
HR (95% CI) ^b^	1	1.35 (090–2.05)	1.18 (0.77–1.81)	1.06 (0.68–1.66)	1.42 (0.93–2.16)	0.32
> 60 years						
HR (95% CI) ^b^	1	0.83 (0.60–1.15)	0.86 (0.63–1.19)	0.86 (0.63–1.18)	0.77 (0.55–1.08)	0.20
**BMI**						
< 30						
HR (95% CI) ^b^	1	1.09 (0.82–1.45)	1.01 (0.76–1.35)	0.94 (0.70–1.26)	1.01 (0.75–1.36)	0.71
> 30						
HR (95% CI) ^b^	1	0.71 (0.41–1.24)	0.96 (0.57–1.62)	1.00 (0.59–1.69)	0.97 (0.56–1.69)	0.72
**Flavanones**						
**Cardiovascular Disease**						
No. of deaths	460	388	333	328	322	
Age-adjusted HR (95% CI) ^a^	1	0.92 (0.80–1.05)	0.82 (0.71–1.05)	0.82 (0.71–0.94)	0.82 (0.71–0.95)	0.002
Multivariate-adjusted HR (95% CI) ^b^	1	0.90 (0.78–1.03)	0.82 (0.71–0.94)	0.83 (0.72–0.86)	0.86 (0.73–1.00)	0.02
**Age**						
< 60 years						
HR (95% CI) ^b^	1	0.87 (0.71–1.11)	0.85 (0.69–1.05)	0.75 (0.60–0.94)	0.84 (0.67–1.05)	0.04
> 60 years						
HR (95% CI) ^b^	1	0.92 (0.76–1.11)	0.77 (0.63–0.95)	0.98 (0.80–1.20)	0.94 (0.76–1.17)	0.66
**BMI**						
< 30						
HR (95% CI) ^b^	1	0.87 (0.74–1.01)	0.79 (0.68–0.93)	0.82 (0.70–0.97)	0.86 (0.72–1.03)	0.04
> 30						
HR (95% CI) ^b^	1	1.08 (0.77–1.52)	0.89 (0.63–1.26)	0.94 (0.67–1.32)	0.93 (0.66–1.32)	0.48
**Coronary heart disease**						
No. of deaths	195	176	161	143	160	
Age-adjusted HR (95% CI) ^a^	1	0.97 (0.79–1.19)	0.92 (0.74–1.13)	0.84 (0.67–1.04)	0.95 (0.77–1.17)	0.30
Multivariate-adjusted HR (95% CI) ^b^	1	0.95 (0.77–1.16)	0.88 (0.71–1.10)	0.82 (0.65–1.02)	0.92 (0.73–1.16)	0.22
**Age**						
< 60 years						
HR (95% CI) ^b^	1	0.94 (0.70–1.27)	1.02 (0.76–1.37)	0.81 (0.59–1.12)	1.05 (0.77–1.43)	0.92
> 60 years						
HR (95% CI) ^b^	1	0.97 (0.72–1.29)	0.76 (0.55–1.05)	0.89 (0.65–1.22)	0.86 (0.61–1.20)	0.27
**BMI**						
< 30						
HR (95% CI) ^b^	1	0.98 (0.78–1.23)	0.89 (0.70–1.13)	0.91 (0.71–1.16)	0.98 (0.75–1.26)	0.63
> 30						
HR (95% CI) ^b^	1	0.82 (0.50–1.35)	0.86 (0.53–1.37)	0.56 (0.34–0.94)	0.78 (0.48–1.27)	0.15
**Stroke**						
No. of deaths	158	139	109	115	99	
Age-adjusted HR (95% CI) ^a^	1	0.98 (0.78–1.23)	0.80 (0.63–1.03)	0.86 (0.68–1.10)	0.76 (0.58–0.98)	0.01
Multivariate-adjusted HR (95% CI) ^b^	1	0.94 (0.74–1.18)	0.78 (0.61–1.00)	0.87 (0.68–1.11)	0.80 (0.61–1.06)	0.08
**Age**						
< 60 years						
HR (95% CI) ^b^	1	0.72 (0.49–1.05)	0.67 (0.45–0.99)	0.79 (0.54–1.16)	0.68 (0.44–1.04)	0.14
> 60 years						
HR (95% CI) ^b^	1	1.10 (0.82–1.48)	0.88 (0.63–1.21)	0.98 (0.70–1.36)	0.96 (0.68–1.36)	0.60
**BMI**						
< 30						
HR (95% CI) ^b^	1	0.84 (0.65–1.08)	0.76 (0.58–1.00)	0.76 (0.57–1.01)	0.74 (0.55–1.01)	0.03
> 30						
HR (95% CI) ^b^	1	1.80 (0.98–3.31)	1.09 (0.56–2.13)	1.69 (0.92–3.08)	1.37 (0.72–2.59)	0.52
**Flavones**						
**Cardiovascular Disease**						
No. of deaths	466	359	349	332	325	
Age-adjusted HR (95% CI) ^a^	1	0.84 (0.73–0.97)	0.86 (0.75–0.99)	0.82 (0.71–0.94)	0.81 (0.70–0.93)	0.004
Multivariate-adjusted HR (95% CI) ^b^	1	0.87 (0.76–1.00)	0.89 (0.77–1.03)	0.84 (0.72–0.98)	0.85 (0.72–1.00)	0.04
**Age**						
< 60 years						
HR (95% CI) ^b^	1	0.76 (0.61–0.94)	0.82 (0.66–1.01)	0.87 (0.70–1.08)	0.82 (0.65–1.03)	0.32
> 60 years						
HR (95% CI) ^b^	1	0.95 (0.79–1.14)	0.95 (0.78–1.16)	0.80 (0.64–0.99)	0.92 (0.73–1.14)	0.17
**BMI**						
< 30						
HR (95% CI) ^b^	1	0.89 (0.76–1.04)	0.85 (0.81–1.12)	0.88 (0.74–1.04)	0.86 (0.72–1.03)	0.12
> 30						
HR (95% CI) ^b^	1	0.79 (0.57–1.10)	0.70 (0.50–0.99)	0.75 (0.53–1.04)	0.83 (0.59–1.15)	0.30
**Coronary Heart Disease**						
No. of deaths	186	160	159	171	159	
Age-adjusted HR (95% CI) ^a^	1	0.93 (0.75–1.15)	0.97 (0.78–1.19)	1.03 (0.84–1.28)	0.97 (0.78–1.20)	0.82
Multivariate-adjusted HR (95% CI) ^b^	1	0.96 (0.77–1.19)	0.97 (0.77–1.21)	1.04 (0.83–1.29)	0.97 (0.76–1.23)	0.92
**Age**						
< 60 years						
HR (95% CI) ^b^	1	0.84 (0.61–1.14)	0.86 (0.63–1.17)	1.05 (0.77–1.42)	0.95 (0.68–1.31)	0.68
> 60 years						
HR (95% CI) ^b^	1	1.06 (0.79–1.44)	1.11 (0.81–1.52)	1.00 (0.71–1.39)	1.02 (0.72–1.46)	0.97
**BMI**						
< 30						
HR (95% CI) ^b^	1	0.90 (0.71–1.15)	0.99 (0.78–1.24)	1.09 (0.85–1.39)	0.82 (0.71–1.22)	0.82
> 30						
HR (95% CI) ^b^	1	1.22 (0.75–1.99)	0.93 (0.55–1.57)	0.91 (0.54–1.54)	1.15 (0.69–1.91)	0.98
**Stroke**						
No. of deaths	171	127	121	101	100	
Age-adjusted HR (95% CI) ^a^	1	0.83 (0.66–1.05)	0.85 (0.67–1.08)	0.71 (0.55–0.91)	0.70 (0.55–0.90)	0.002
Multivariate-adjusted HR (95% CI) ^b^	1	0.84 (0.67–1.07)	0.86 (0.67–1.10)	0.71 (0.55–0.93)	0.74 (0.56–0.98)	0.01
**Age**						
< 60 years						
HR (95% CI) ^b^	1	0.63 (0.42–0.94)	0.77 (0.52–1.13)	0.66 (0.44–0.99)	0.63 (0.41–0.98)	0.07
> 60 years						
HR (95% CI) ^b^	1	0.97 (0.72–1.30)	0.90 (0.66–1.24)	0.74 (0.52–1.05)	0.84 (0.59–1.21)	0.14
**BMI**						
< 30						
HR (95% CI) ^b^	1	0.92 (0.71–1.20)	0.96 (0.73–1.26)	0.75 (0.56–1.02)	0.74 (0.54–1.03)	0.03
> 30						
HR (95% CI) ^b^	1	0.58 (0.33–1.01)	0.59 (0.34–1.03)	0.60 (0.34–1.04)	0.70 (0.40–1.20)	0.29
**Isoflavnoids**						
**Cardiovascular Disease**						
No. of deaths	438	396	350	326	321	
Age-adjusted HR (95% CI) ^a^	1	0.96 (0.84–1.10)	0.89 (0.77–1.03)	0.88 (0.76–1.01)	0.89 (0.77–1.03)	0.05
Multivariate-adjusted HR (95% CI) ^b^	1	0.96 (0.84–1.10)	0.91 (0.79–1.05)	0.91 (0.79–1.06)	0.96 (0.82–1.11)	0.42
**Age**						
< 60 years						
HR (95% CI) ^b^	1	1.06 (0.87–1.31)	0.96 (0.77–1.19)	0.90 (0.72–1.12)	0.95 (0.76–1.19)	0.30
> 60 years						
HR (95% CI) ^b^	1	0.86 (0.72–1.04)	0.86 (0.71–1.05)	0.95 (0.77–1.16)	0.95 (0.77–1.16)	0.78
**BMI**						
< 30						
HR (95% CI) ^b^	1	0.89 (0.77–1.04)	0.88 (0.75–1.04)	0.90 (0.77–1.07)	0.94 (0.80–1.12)	0.53
> 30						
HR (95% CI) ^b^	1	1.27 (0.92–1.74)	1.05 (0.76–1.46)	0.98 (0.70–1.37)	1.03 (0.72–1.47)	0.58
**Coronary Heart Disease**						
No. of deaths	186	179	174	155	141	
Age-adjusted HR (95% CI) ^a^	1	1.01 (0.82–1.24)	1.03 (0.83–1.27)	0.96 (0.78–1.19)	0.90 (0.72–1.12)	0.34
Multivariate-adjusted HR (95% CI) ^b^	1	1.00 (0.81–1.23)	1.02 (0.82–1.26)	0.95 (0.76–1.19)	0.90 (0.72–1.13)	0.36
**Age**						
< 60 years						
HR (95% CI) ^b^	1	1.08 (0.81–1.45)	1.01 (0.74–1.36)	0.88 (0.65–1.21)	0.94 (0.69–1.29)	0.36
> 60 years						
HR (95% CI) ^b^	1	0.90 (0.67–1.22)	1.02 (0.75–1.38)	1.05 (0.76–1.43)	0.82 (0.59–1.16)	0.61
**BMI**						
< 30						
HR (95% CI) ^b^	1	0.96 (0.76–1.21)	1.01 (0.80–1.28)	0.93 (0.73–1.20)	0.87 (0.68–1.13)	0.34
> 30						
HR (95% CI) ^b^	1	1.20 (0.74–1.94)	1.09 (0.67–1.78)	1.05 (0.65–1.72)	1.03 (0.60–1.73)	0.90
**Stroke**						
No. of deaths	155	144	104	105	112	
Age-adjusted HR (95% CI) ^a^	1	1.01 (0.80–1.27)	0.77 (0.60–0.99)	0.83 (0.65–1.06)	0.92 (0.72–1.18)	0.96
Multivariate-adjusted HR (95% CI) ^b^	1	0.99 (0.79–1.25)	0.78 (0.61–1.01)	0.88 (0.68–1.14)	1.04 (0.81–1.34)	0.78
**Age**						
< 60 years						
HR (95% CI) ^b^	1	1.22 (0.83–1.80)	1.03 (0.68–1.50)	1.04 (0.69–1.57)	0.96 (0.62–1.48)	0.61
> 60 years						
HR (95% CI) ^b^	1	0.87 (0.65–1.17)	0.65 (0.47–0.91)	0.80 (0.58–1.12)	1.10 (0.81–1.51)	1.00
**BMI**						
< 30						
HR (95% CI) ^b^	1	0.87 (0.67–1.13)	0.75 (0.56–1.00)	0.84 (0.63–1.12)	1.00 (0.76–1.33)	0.77
> 30						
HR (95% CI) ^b^	1	1.69 (1.00–2.86)	1.01 (0.56–1.82)	1.18 (0.66–2.10)	1.31 (0.71–2.44)	0.91
**Anthocianidins**						
**Cardiovascular Disease**						
No. of deaths	403	326	350	362	390	
Age-adjusted HR (95% CI) ^a^	1	0.85 (0.73–0.98)	0.90 (0.78–1.04)	0.91 (0.79–1.05)	1.02 (0.88–1.17)	0.53
Multivariate-adjusted HR (95% CI) ^b^	1	0.86 (0.75–1.00)	0.92 (0.79–1.06)	0.92 (0.79–1.06)	1.01 (0.87–1.17)	0.67
**Age**						
< 60 years						
HR (95% CI) ^b^	1	0.90 (0.73–1.11)	0.76 (0.61–0.94)	0.79 (0.64–0.97)	0.76 (0.62–0.93)	0.32
> 60 years						
HR (95% CI) ^b^	1	0.84 (0.68–1.02)	0.89 (0.73–1.09)	0.89 (0.73–1.08)	0.77 (0.62–0.95)	0.059
**BMI**						
< 30						
HR (95% CI) ^b^	1	0.89 (0.75–1.05)	0.91 (0.77–1.07)	0.87 (0.74–1.03)	0.99 (0.84–1.17)	0.85
> 30						
HR (95% CI) ^b^	1	0.74 (0.53–1.01)	0.86 (0.63–1.17)	0.93 (0.68–1.27)	0.79 (0.56–1.10)	0.46
**Coronary Heart Disease**						
No. of deaths	192	133	159	169	182	
Age-adjusted HR (95% CI) ^a^	1	0.72 (0.57–0.89)	0.85 (0.69–1.05)	0.88 (0.72–1.09)	0.98 (0.80–1.20)	0.56
Multivariate-adjusted HR (95% CI) ^b^	1	0.71 (0.57–0.89)	0.83 (0.67–1.03)	0.85 (0.69–1.05)	0.92 (0.74–1.14)	0.90
**Age**						
< 60 years						
HR (95% CI) ^b^	1	0.72 (0.53–0.99)	0.82 (0.60–1.11)	0.90 (0.67–1.22)	1.02 (0.76–1.37)	0.40
> 60 years						
HR (95% CI) ^b^	1	0.66 (0.48–0.92)	0.80 (0.59–1.09)	0.72 (0.53–0.98)	0.68 (0.49–0.94)	0.04
**BMI**						
< 30						
HR (95% CI) ^b^	1	0.72 (0.56–0.93)	0.85 (0.67–1.09)	0.79 (0.62–1.01)	0.86 (0.67–1.09)	0.39
> 30						
HR (95% CI) ^b^	1	0.65 (0.40–1.04)	0.68 (0.42–1.09)	0.94 (0.60–1.47)	0.87 (0.55–1.40)	0.98
**Stroke**						
No. of deaths	142	120	115	113	130	
Age-adjusted HR (95% CI) ^a^	1	0.91 (0.71–1.16)	0.86 (0.67–1.11)	0.83 (0.64–1.16)	0.99 (0.78–1.26)	0.67
Multivariate-adjusted HR (95% CI) ^b^	1	0.96 (0.75–1.22)	0.91 (0.71–1.17)	0.86 (0.66–1.11)	1.-04 (0.81–1.34)	0.91
**Age**						
< 60 years						
HR (95% CI) ^b^	1	1.02 (0.69–1.50)	0.89 (0.59–1.34)	0.71 (0.45–1.10)	1.21 (0.81–1.80)	0.80
> 60 years						
HR (95% CI) ^b^	1	0.88 (0.64–1.21)	0.87 (0.63–1.19)	0.88 (0.64–1.21)	0.83 (0.59–1.17)	0.34
**BMI**						
< 30						
HR (95% CI) ^b^	1	1.04 (0.79–1.37)	0.88 (0.66–1.17)	0.78 (0.58–1.05)	1.04 (0.78–1.39)	0.57
> 30						
HR (95% CI) ^b^	1	0.64 (0.36–1.14)	0.98 (0.59–1.64)	1.08 (0.65–1.80)	0.89 (0.51–1.56)	0.74
**Dihydrochalcones**						
**Cardiovascular Disease**						
No. of deaths	762		374	381	314	
Age-adjusted HR (95% CI) ^a^	1		1.01 (0.89–1.14)	1.00 (0.88–1.13)	0.83 (0.73–0.95)	0.02
Multivariate-adjusted HR (95% CI) ^b^	1		1.01 (0.89–1.14)	0.83 (0.73–0.95)	0.86 (0.75–0.99)	0.10
**Age**						
< 60 years						
HR (95% CI) ^b^	1		0.95 (0.79–1.14)	1.08 (0.90–1.29)	0.96 (0.80–1.17)	0.91
> 60 years						
HR (95% CI) ^b^	1		1.05 (0.88–1.24)	0.99 (0.83–1.18)	0.81 (0.67–0.98)	0.07
**BMI**						
< 30						
HR (95% CI) ^b^	1		1.04 (0.91–1.20)	1.05 (0.91–1.20)	0.87 (0.74–1.02)	0.25
> 30						
HR (95% CI) ^b^	1		0.82 (0.61–1.10)	0.91 (0.69–1.20)	0.79 (0.60–1.04)	0.13
**Coronary Heart Disease**						
No. of deaths	335		159	187	154	
Age-adjusted HR (95% CI) ^a^	1		0.97 (0.93–1.33)	1.11 (0.93–1.33)	0.93 (0.77–1.12)	0.90
Multivariate-adjusted HR (95% CI) ^b^	1		0.96 (0.80–1.17)	1.11 (0.92–1.33)	0.93 (0.76–1.13)	0.90
**Age**						
< 60 years						
HR (95% CI) ^b^	1		0.95 (0.72–1.23)	1.16 (0.90–1.49)	1.09 (0.84–1.42)	0.27
> 60 years						
HR (95% CI) ^b^	1		0.98 (0.74–1.28)	1.11 (0.85–1.45)	0.79 (0.58–1.07)	0.36
**BMI**						
< 30						
HR (95% CI) ^b^	1		1.03 (0.84–1.27)	1.16 (0.95–1.42)	0.88 (0.70–1.10)	0.76
> 30						
HR (95% CI) ^b^	1		0.67 (0.42–1.07)	0.96 (0.64–1.44)	1.02 (0.69–1.49)	0.80
**Stroke**						
No. of deaths	270		139	130	91	
Age-adjusted HR (95% CI) ^a^	1		0.99 (0.80–1.23)	0.97 (0.78–1.19)	0.68 (0.54–0.87)	0.008
Multivariate-adjusted HR (95% CI) ^b^	1		0.98 (0.79–1.21)	0.96 (0.78–1.19)	0.71 (0.56–0.91)	0.02
**Age**						
< 60 years						
HR (95% CI) ^b^	1		0.81 (0.57–1.16)	1.14 (0.83–1.57)	0.76 (0.52–1.12)	0.49
> 60 years						
HR (95% CI) ^b^	1		1.09 (0.84–1.42)	0.88 (0.66–1.17)	0.71 (0.52–0.98)	0.04
**BMI**						
< 30						
HR (95% CI) ^b^	1		0.88 (0.71–1.00)	0.95 (0.75–1.21)	0.70 (0.53–0.93)	0.03
> 30						
HR (95% CI) ^b^	1		1.38 (0.88–1.53)	1.10 (0.69–1.76)	0.76 (0.45–1.27)	0.36

As shown in Table 4, after adjusting for confounding variables, participants with higher isoflavonoids and dihydrochalcones intakes had a lower risk of cancer mortality (HR: 0.82; CI:0.68–0.98 and HR: 0.84; CI: 0.71–0.99). Isoflavonoids intake had also a protective role regarding GI cancers mortality (HR: 0.80; CI: 0.61–1.04) especially in young and obese participants (*p* for trends = 0.003 and 0.01, respectively).

**Table 4:** Association between dietary flavonoids intake and cancer mortality

Flavonoids	Quintile of intake					*p* for trend
	1	2	3	4	5	
**Total flavonoids**						
**cancer**						
No. of deaths	186	223	235	243	265	
Age-adjusted HR (95% CI) ^a^	1	1.22 (1.00–1.48)	1.27 (1.05–1.54)	1.27 (1.05–1.54)	1.43 (1.19–1.73)	< 0.001
Multivariate-adjusted HR (95% CI) ^b^	1	1.18 (0.97–1.44)	1.17 (0.96–1.42)	1.13 (0.93–1.38)	1.19 (0.97–1.44)	0.19
**Age**						
< 60 years						
HR (95% CI) ^b^	1	1.03 (0.79–1.35)	1.07 (0.82–1.38)	1.11 (0.85–1.44)	1.04 (0.80–1.35)	0.64
> 60 years						
HR (95% CI) ^b^	1	1.36 (1.01–1.82)	1.19 (0.89–1.60)	1.08 (0.80–1.46)	1.23 (0.91–1.65)	0.62
**BMI**						
< 30						
HR (95% CI) ^b^	1	1.08 (0.85–1.35)	1.12 (0.90–1.38)	1.11 (0.89–1.37)	1.13 (0.91–1.40)	0.28
> 30						
HR (95% CI) ^b^	1	1.66 (1.07–3.79)	1.41 (0.88–2.23)	1.14 (0.70–1.97)	1.28 (0.78–2.11)	0.86
**GI cancer**						
No. of deaths	108	123	123	145	143	
Age-adjusted HR (95% CI) ^a^	1	1.17 (0.90–1.52)	1.16 (0.90–1.51)	1.32 (1.02–1.69)	1.35 (1.05–1.73)	0.01
Multivariate-adjusted HR (95% CI) ^b^	1	1.12 (0.86–1.45)	1.02 (0.78–1.32)	1.11 (0.86–1.44)	1.04 (0.80–1.35)	0.80
**Age**						
< 60 years						
HR (95% CI) ^b^	1	0.85 (0.59–1.24)	0.97 (0.68–1.38)	1.04 (0.73–1.48)	0.80 (0.55–1.15)	0.53
> 60 years						
HR (95% CI) ^b^	1	1.41 (0.98–2.04)	0.96 (0.65–1.42)	1.09 (0.75–1.59)	1.20 (0.82–1.74)	0.81
**BMI**						
< 30						
HR (95% CI) ^b^	1	1.00 (0.85–1.35)	0.97 (0.73–1.28)	1.06 (0.81–1.40)	0.99 (0.74–1.31)	0.89
> 30						
HR (95% CI) ^b^	1	2.01 (1.06–3.79)	1.36 (0.68–2.71)	1.35 (0.67–2.70)	1.26 (0.61–2.60)	0.98
**Other cancer**						
No. of deaths	78	100	112	98	122	
Age-adjusted HR (95% CI) ^a^	1	1.28 (0.95–1.73)	1.42 (1.07–1.90)	1.21 (0.90–1.63)	1.55 (1.17–2.07)	0.01
Multivariate-adjusted HR (95% CI) ^b^	1	1.27 (0.99–1.71)	1.38 (1.03–1.85)	1.14 (0.84–1.55)	1.39 (1.53–1.88)	0.10
**Age**						
< 60 years						
HR (95% CI) ^b^	1	1.25 (0.85–1.83)	1.78 (0.80–1.73)	1.17 (0.79–1.73)	1.36 (0.93–2.00)	0.20
> 60 years						
HR (95% CI) ^b^	1	1.27 (0.78–2.05)	1.58 (1.00–2.49)	1.05 (0.63–1.71)	1.27 (0.78–2.06)	0.63
**BMI**						
< 30						
HR (95% CI) ^b^	1	1.22 (0.87–1.72)	1.35 (0.97–1.88)	1.17 (0.83–1.64)	1.36 (0.97–1.90)	0.14
> 30						
HR (95% CI) ^b^	1	1.40 (0.75–2.59)	1.44 (0.77–2.69)	0.96 (0.48–1.93)	1.32 (0.67–2.61)	0.80
**Flavonols**						
**Cancer**						
No. of deaths	186	226	223	253	265	
Age-adjusted HR (95% CI) ^a^	1	1.23 (1.01–1.49)	1.99 (0.98–1.45)	1.31 (1.08–1.58)	1.42 (1.18–1.71)	< 0.001
Multivariate-adjusted HR (95% CI) ^b^	1	1.21 (0.99–1.47)	1.11 (0.91–1.36)	1.17 (0.96–1.42)	1.19 (0.97–1.45)	0.18
**Age**						
< 60 years						
HR (95% CI) ^b^	1	0.97 (0.74–1.26)	0.98 (0.76–1.28)	1.12 (0.87–1.44)	0.96 (0.74–1.26)	0.80
> 60 years						
HR (95% CI) ^b^	1	1.54 (1.15–2.07)	1.21 (0.89–1.64)	1.15 (0.85–1.55)	1.35 (1.00–1.83)	0.42
**BMI**						
< 30						
HR (95% CI) ^b^	1	1.15 (0.75–1.34)	1.04 (0.83–1.29)	1.17 (0.94–1.45)	1.15 (0.93–1.43)	0.22
> 30						
HR (95% CI) ^b^	1	1.46 (0.93–2.27)	1.48 (0.94–2.31)	1.04 (0.63–1.71)	1.16 (0.70–1.91)	0.94
**GI cancer**						
No. of deaths	109	120	120	151	143	
Age-adjusted HR (95% CI) ^a^	1	1.12 (0.87–1.46)	1.11 (0.86–1.44)	1.34 (1.05–1.71)	1.32 (1.03–1.70)	0.009
Multivariate-adjusted HR (95% CI) ^b^	1	1.10 (0.76–1.29)	0.99 (0.76–1.29)	1.14 (0.88–1.47)	1.03 (0.79–1.35)	0.71
**Age**						
< 60 years						
HR (95% CI) ^b^	1	0.77 (0.53–1.11)	0.88 (0.60–1.23)	1.10 (0.79–1.54)	0.69 (0.48–1.00)	0.42
> 60 years						
HR (95% CI) ^b^	1	1.54 (1.06)	1.10 (0.74–1.63)	1.07 (0.74–1.58)	1.38 (0.94–2.02)	0.53
**BMI**						
< 30						
HR (95% CI) ^b^	1	1.00 (0.75–1.34)	0.92 (0.69–1.23)	1.13 (0.86–1.49)	1.01 (0.76–1.34)	0.61
> 30						
HR (95% CI) ^b^	1	1.74 (0.93–3.27)	1.51 (0.79–2.89)	1.06 (0.52–2.16)	1.04 (0.50–2.15)	0.58
**Other cancer**						
No. of deaths	77	106	103	102	122	
Age-adjusted HR (95% CI) ^a^	1	1.37 (1.02–1.84)	1.31 (0.98–1.77)	1.26 (0.94–1.70)	1.56 (1.17–2.08)	0.01
Multivariate-adjusted HR (95% CI) ^b^	1	1.37 (1.01–1.84)	1.28 (0.95–1.72)	1.20 (0.88–1.62)	1.40 (1.04–1.90)	0.13
**Age**						
< 60 years						
HR (95% CI) ^b^	1	1.23 (0.84–1.80)	1.15 (0.78–1.68)	1.11 (0.75–1.64)	1.35 (0.92–1.97)	0.25
> 60 years						
HR (95% CI) ^b^	1	1.56 (0.97–2.51)	1.40 (0.86–2.27)	1.27 (0.78–2.07)	1.31 (0.80–2.16)	0.63
**BMI**						
< 30						
HR (95% CI) ^b^	1	1.39 (0.99–1.95)	1.23 (0.87–1.73)	1.22 (0.86–1.71)	1.38 (0.98–1.94)	0.21
> 30						
HR (95% CI) ^b^	1	1.23 (0.65–2.30)	1.43 (0.77–2.66)	1.03 (0.51–2.05)	1.30 (0.65–2.59)	0.64
**Flavan-3-ol monomers**						
**Cancer**						
No. of deaths	186	218	234	246	269	
Age-adjusted HR (95% CI) ^a^	1	1.17 (0.96–1.42)	1.27 (1.05–1.54)	1.26 (1.04–1.52)	1.44 (1.19–1.73)	< 0.001
Multivariate-adjusted HR (95% CI) ^b^	1	1.40 (0.93–1.39)	1.16 (0.95–1.41)	1.12 (0.92–1.36)	1.19 (0.98–1.44)	0.14
**Age**						
< 60 years						
HR (95% CI) ^b^	1	1.01 (0.77–1.320	1.08 (0.83–1.39)	1.07 (0.82–1.38)	1.04 (0.80–1.35)	0.66
> 60 years						
HR (95% CI) ^b^	1	1.30 (0.97–1.75)	1.15 (0.85–1.55)	1.12 (0.83–1.50)	1.23 (0.92–1.66)	0.45
**BMI**						
< 30						
HR (95% CI) ^b^	1	1.02 (0.82–1.28)	1.08 (0.87–1.33)	1.09 (0.88–1.34)	1.11 (0.90–1.38)	0.25
> 30						
HR (95% CI) ^b^	1	1.73 (1.11–2.71)	1.58 (0.99–2.51)	1.18 (0.71–1.94)	1.40 (0.85–2.30)	0.67
**GI cancer**						
No. of deaths	108	115	127	148	145	
Age-adjusted HR (95% CI) ^a^	1	1.07 (0.82–1.39)	1.21 (0.83–1.56)	1.31 (1.02–1.68)	1.35 (1.05–1.73)	0.004
Multivariate-adjusted HR (95% CI) ^b^	1	1.03 (0.79–1.34)	1.04 (0.80–1.35)	1.11 (0.86–1.43)	1.03 (0.80–1.34)	0.62
**Age**						
< 60 years						
HR (95% CI) ^b^	1	0.78 (0.53–1.14)	0.99 (0.70–1.40)	1.02 (0.72–1.44)	0.79 (0.55–1.14)	0.61
> 60 years						
HR (95% CI) ^b^	1	1.34 (0.92–1.95)	0.98 (0.67–1.45)	1.13 (0.78–1.64)	0.63 (0.83–1.75)	0.63
**BMI**						
< 30						
HR (95% CI) ^b^	1	0.91 (0.68–1.21)	0.95 (0.72–1.26)	1.05 (0.80–1.38)	0.96 (0.73–1.27)	0.79
> 30						
HR (95% CI) ^b^	1	1.98 (1.03–3.80)	1.67 (0.84–3.30)	1.41 (0.69–2.87)	1.42 (0.69–2.94)	0.73
**Other cancer**						
No. of deaths	78	103	107	98	124	
Age-adjusted HR (95% CI) ^a^	1	1.31 (0.97–1.75)	1.36 (1.02–1.83)	1.19 (0.88–1.60)	1.57 (1.18–2.08)	0.01
Multivariate-adjusted HR (95% CI) ^b^	1	1.29 (0.96–1.74)	1.32 (0.96–1.74)	1.12 (0.83–1.52)	1.40 (1.04–1.89)	0.11
**Age**						
< 60 years						
HR (95% CI) ^b^	1	1.30 (0.88–1.90)	1.18 (0.80–1.73)	1.10 (0.75–1.64)	1.37 (0.94–2.01)	0.27
> 60 years						
HR (95% CI) ^b^	1	1.29 (0.78–2.02)	1.43 (0.90–2.27)	1.09 (0.68–1.77)	1.28 (0.79–2.07)	0.55
**BMI**						
< 30						
HR (95% CI) ^b^	1	1.21 (0.86–1.70)	1.27 (0.91–1.77)	1.13 (0.81–2.59)	1.35 (0.97–1.89)	0.15
> 30						
HR (95% CI) ^b^	1	1.55 (0.84–2.87)	1.50 (0.79–2.82)	0.97 (0.47–1.99)	1.39 (0.70–2.77)	0.80
**Flavanones**						
**Cancer**						
No. of deaths	287	238	231	196	200	
Age-adjusted HR (95% CI) ^a^	1	0.88 (0.74–1.00)	0.89 (0.74–1.06)	0.77 (0.64–0.92)	0.80 (0.66–0.95)	0.004
Multivariate-adjusted HR (95% CI) ^b^	1	0.87 (0.73–1.04)	0.88 (0.74–1.05)	0.78 (0.65–0.94)	0.85 (0.70–1.03)	0.04
**Age**						
< 60 years						
HR (95% CI) ^b^	1	0.81 (0.64–1.04)	0.90 (0.71–1.14)	0.86 (0.67–1.10)	0.89 (0.69–1.16)	0.54
> 60 years						
HR (95% CI) ^b^	1	0.93 (0.73–1.20)	0.88 (0.68–1.15)	0.74 (0.55–0.99)	0.85 (0.63–1.14)	0.09
**BMI**						
< 30						
HR (95% CI) ^b^	1	0.92 (0.76–1.10)	0.91 (0.75–1.10)	0.79 (0.64–0.98)	0.88 (0.71–1.09)	0.09
> 30						
HR (95% CI) ^b^	1	0.55 (0.34–0.88)	0.64 (0.42–1.00)	0.58 (0.38–0.91)	0.55 (0.35–0.86)	0.02
**GI cancer**						
No. of deaths	154	148	132	109	99	
Age-adjusted HR (95% CI) ^a^	1	1.04 (0.83–1.30)	0.96 (0.76–1.22)	0.81 (0.64–1.04)	0.75 (0.58–0.97)	0.007
Multivariate-adjusted HR (95% CI) ^b^	1	1.02 (0.81–1.28)	0.96 (0.76–1.22)	0.84 (0.65–1.08)	0.85 (0.65–1.11)	0.09
**Age**						
< 60 years						
HR (95% CI) ^b^	1	0.83 (0.59–1.18)	1.08 (0.78–1.50)	0.88 (0.62–1.26)	0.96 (0.66–1.39)	0.23
> 60 years						
HR (95% CI) ^b^	1	1.22 (0.90–1.66)	0.85 (0.60–1.21)	0.84 (0.58–1.22)	0.72 (0.51–1.13)	0.04
**BMI**						
< 30						
HR (95% CI) ^b^	1	1.05 (0.83–1.34)	0.91 (0.70–1.17)	0.79 (0.59–1.04)	0.80 (0.60–1.08)	0.02
> 30						
HR (95% CI) ^b^	1	0.61 (0.28–1.33)	1.07 (0.56–2.04)	0.88 (0.46–1.71)	0.80 (0.40–1.60)	0.86
**Other cancer**						
No. of deaths	133	90	99	87	101	
Age-adjusted HR (95% CI) ^a^	1	0.71 (0.54–0.93)	0.80 (0.62–1.04)	0.72 (0.55–0.95)	0.84 (0.65–1.09)	0.22
Multivariate-adjusted HR (95% CI) ^b^	1	0.70 (0.53–0.91)	0.79 (0.61–1.04)	0.72 (0.55–0.96)	0.83 (0.63–1.11)	0.24
**Age**						
< 60 years						
HR (95% CI) ^b^	1	0.79 (0.56–1.12)	0.73 (0.51–1.04)	0.84 (0.59–1.19)	0.82 (0.56–1.19)	0.38
> 60 years						
HR (95% CI) ^b^	1	0.54 (0.34–0.85)	0.92 (0.62–1.38)	0.60 (0.38–0.97)	0.96 (0.62–1.48)	0.84
**BMI**						
< 30						
HR (95% CI) ^b^	1	0.73 (0.54–0.99)	0.91 (0.68–1.22)	0.81 (0.59–1.11)	0.97 (0.71–1.33)	0.97
> 30						
HR (95% CI) ^b^	1	0.51 (0.28–0.93)	0.39 (0.21–0.74)	0.41 (0.22–0.76)	0.41 (0.22–0.76)	0.004
**Flavones**						
**Cancer**						
No. of deaths	285	225	225	199	219	
Age-adjusted HR (95% CI) ^a^	1	0.84 (0.71–1.00)	0.88 (0.74–1.05)	0.77 (0.65–0.93)	0.86 (0.72–1.03)	0.06
Multivariate-adjusted HR (95% CI) ^b^	1	0.82 (0.69–0.98)	0.84 (0.70–1.01)	0.74 (0.61–0.90)	0.85 (0.70–1.04)	0.05
**Age**						
< 60 years						
HR (95% CI) ^b^	1	0.86 (0.67–1.09)	0.81 (0.63–1.04)	0.73 (0.56–0.94)	0.81 (0.62–1.06)	0.06
> 60 years						
HR (95% CI) ^b^	1	0.76 (0.58–0.99)	0.87 (0.67–1.14)	0.76 (0.57–1.02)	0.95 (0.71–1.27)	0.66
**BMI**						
< 30						
HR (95% CI) ^b^	1	0.83 (0.69–1.01)	0.90 (0.74–1.10)	0.71 (0.57–0.88)	0.90 (0.72–1.12)	0.13
> 30						
HR (95% CI) ^b^	1	0.74 (0.47–1.16)	0.54 (0.33–0.87)	0.76 (0.49–1.18)	0.58 (0.36–0.93)	0.06
**GI cancer**						
No. of deaths	159	130	125	116	113	
Age-adjusted HR (95% CI) ^a^	1	0.89 (0.70–1.12)	0.90 (0.71–1.14)	0.83 (0.65–1.06)	0.82 (0.64(−1.05)	0.09
Multivariate-adjusted HR (95% CI) ^b^	1	0.86 (0.68–1.09)	0.85 (0.66–1.08)	0.79 (0.61–1.02)	0.84 (0.64–1.10)	0.15
**Age**						
< 60 years						
HR (95% CI) ^b^	1	0.94 (0.67–1.31)	0.78 (0.55–1.11)	0.73 (0.51–1.06)	0.79 (0.54–1.16)	0.09
> 60 years						
HR (95% CI) ^b^	1	0.76 (0.54–1.08)	0.92 (0.65–1.29)	0.85 (0.59–1.22)	0.90 (0.61–1.32)	0.73
**BMI**						
< 30						
HR (95% CI) ^b^	1	0.85 (0.66–1.09)	0.90 (0.69–1.16)	0.76 (0.57–1.01)	0.86 (0.64–1.16)	0.21
> 30						
HR (95% CI) ^b^	1	0.91 (0.48–1.71)	0.56 (0.28–1.14)	0.81 (0.43–1.53)	0.59 (0.30–1.17)	0.14
**Other cancer**						
No. of deaths	126	95	100	83	106	
Age-adjusted HR (95% CI) ^a^	1	0.79 (0.60–1.03)	0.86 (0.66–1.12)	0.70 (0.53–0.93)	0.91 (0.70–1.19)	0.33
Multivariate-adjusted HR (95% CI) ^b^	1	0.77 (0.59–1.02)	0.83 (0.63–1.09)	0.68 (0.50–0.91)	0.86 (0.64–1.16)	0.21
**Age**						
< 60 years						
HR (95% CI) ^b^	1	0.78 (0.54–1.11)	0.84 (0.59–1.19)	0.71 (0.49–1.04)	0.82 (0.56–1.21)	0.30
> 60 years						
HR (95% CI) ^b^	1	0.75 (0.49–1.15)	0.80 (0.52–1.25)	0.63 (0.39–1.03)	1.02 (0.65–1.60)	0.77
**BMI**						
< 30						
HR (95% CI) ^b^	1	0.82 (0.61–1.11)	0.92 (0.68–1.24)	0.64 (0.46–0.90)	0.95 (0.68–1.31)	0.38
> 30						
HR (95% CI) ^b^	1	0.59 (0.31–1.13)	0.52 (0.26–1.00)	0.73 (0.40–1.33)	0.57 (0.30–1.11)	0.24
**Isoflavnoids**						
**Cancer**						
No. of deaths	287	272	208	196	190	
Age-adjusted HR (95% CI) ^a^	1	0.99 (0.84–1.17)	0.79 (0.66–0.94)	0.78 (0.65–0.93)	0.77 (0.64–0.93)	< 0.001
Multivariate-adjusted HR (95% CI) ^b^	1	0.96 (0.81–1.14)	0.77 (0.64–0.93)	0.79 (0.66–0.96)	0.82 (0.68–0.98)	0.006
**Age**						
< 60 years						
HR (95% CI) ^b^	1	0.97 (0.77–1.22)	0.72 (0.56–0.92)	0.76 (0.59–0.97)	0.73 (0.56–0.94)	0.002
> 60 years						
HR (95% CI) ^b^	1	0.93 (0.72–1.19)	0.83 (0.63–1.08)	0.81 (0.61–1.08)	0.90 (0.67–1.19)	0.22
**BMI**						
< 30						
HR (95% CI) ^b^	1	0.99 (0.83–1.19)	0.81 (0.66–0.99)	0.81 (0.66–1.00)	0.85 (0.65–1.05)	0.02
> 30						
HR (95% CI) ^b^	1	0.72 (0.47–1.10)	0.55 (0.35–0.86)	0.60 (0.39–0.94)	0.60 (0.38–0.95)	0.02
**GI cancer**						
No. of deaths	159	162	126	103	93	
Age-adjusted HR (95% CI) ^a^	1	1.08 (0.87–1.34)	0.88 (0.69–1.11)	0.76 (0.59–0.97)	0.70 (0.54–0.91)	< 0.001
Multivariate-adjusted HR (95% CI) ^b^	1	1.03 (0.83–1.29)	0.87 (0.69–1.11)	0.81 (0.63–1.04)	0.80 (0.61–1.04)	0.02
**Age**						
< 60 years						
HR (95% CI) ^b^	1	1.03 (0.76–1.40)	0.72 (0.51–1.02)	0.74 (0.52–1.05)	0.64 (0.44–0.93)	0.003
> 60 years						
HR (95% CI) ^b^	1	1.02 (0.74–1.04)	1.01 (0.72–1.41)	0.84 (0.58–1.22)	0.96 (0.66–1.39)	0.53
**BMI**						
< 30						
HR (95% CI) ^b^	1	1.07 (0.84–1.36)	0.92 (0.71–1.19)	0.84 (0.63–1.11)	0.86 (0.65–1.15)	0.10
> 30						
HR (95% CI) ^b^	1	0.74 (0.41–1.32)	0.55 (0.30–1.02)	0.54 (0.29–1.01)	0.46 (0.23–0.92)	0.01
**Other cancer**						
No. of deaths	128	110	82	93	97	
Age-adjusted HR (95% CI) ^a^	1	0.88 (0.68–1.14)	0.68 (0.51–0.89)	0.80 (0.61–1.04)	0.86 (0.66–1.12)	0.15
Multivariate-adjusted HR (95% CI) ^b^	1	0.87 (0.67–1.12)	0.66 (0.49–0.88)	0.58 (0.58–1.01)	0.63 (0.63–1.10)	0.10
**Age**						
< 60 years						
HR (95% CI) ^b^	1	0.90 (0.64–1.27)	0.71 (0.49–1.03)	0.77 (0.54–1.11)	0.81 (0.57–1.16)	0.17
> 60 years						
HR (95% CI) ^b^	1	0.81 (0.54–1.21)	0.58 (0.37–0.91)	0.77 (0.49–1.19)	0.81 (0.52–1.25)	0.24
**BMI**						
< 30						
HR (95% CI) ^b^	1	0.89 (0.67–1.19)	0.68 (0.49–0.93)	0.77 (0.56–1.05)	0.84 (0.61–1.13)	0.12
> 30						
HR (95% CI) ^b^	1	0.70 (0.37–1.30)	0.55 (0.29–1.06)	0.68 (0.37–1.26)	0.76 (0.40–1.42)	0.42
**Anthocianidins**						
**Cancer**						
No. of deaths	202	210	237	247	257	
Age-adjusted HR (95% CI) ^a^	1	1.07 (0.88–1.30)	1.20 (0.99–1.45)	1.22 (1.01–1.47)	1.31 (1.09–1.58)	0.001
Multivariate-adjusted HR (95% CI) ^b^	1	1.05 (0.86–1.27)	1.13 (0.93–1.36)	1.09 (0.90–1.32)	1.12 (0.92–1.36)	0.23
**Age**						
< 60 years						
HR (95% CI) ^b^	1	0.89 (0.68–1.16)	1.03 (0.80–1.33)	1.01 (0.78–1.30)	1.00 (0.77–1.30)	0.64
> 60 years						
HR (95% CI) ^b^	1	1.20 (0.90–1.60)	1.15 (0.87–1.54)	1.09 (0.81–1.45)	1.11 (0.83–1.50)	0.73
**BMI**						
< 30						
HR (95% CI) ^b^	1	1.02 (0.82–1.27)	1.09 (0.88–1.35)	1.09 (0.88–1.35)	1.10 (0.89–1.21)	0.27
> 30						
HR (95% CI) ^b^	1	1.00 (0.62–1.62)	1.17 (0.74–1.87)	1.26 (0.80–1.99)	0.99 (0.62–1.60)	0.77
**GI cancer**						
No. of deaths	121	111	127	151	133	
Age-adjusted HR (95% CI) ^a^	1	0.96 (0.74–1.24)	1.09 (0.85–1.40)	1.26 (0.99–1.61)	1.15 (0.90–1.47)	0.04
Multivariate-adjusted HR (95% CI) ^b^	1	0.93 (0.72–1.21)	0.99 (0.77–1.28)	1.06 (0.83–1.36)	0.93 (0.72–1.21)	0.99
**Age**						
< 60 years						
HR (95% CI) ^b^	1	0.76 (0.53–1.10)	0.96 (0.68–1.36)	0.94 (0.67–1.33)	0.73 (0.50–1.05)	0.31
> 60 years						
HR (95% CI) ^b^	1	1.08 (0.75–1.55)	0.93 (0.64–1.35)	1.08 (0.76–1.55)	1.04 (0.72–1.51)	0.80
**BMI**						
< 30						
HR (95% CI) ^b^	1	0.88 (0.66–1.17)	0.96 (0.73–1.27)	1.04 (0.80–1.36)	0.92 (0.69–1.21)	0.99
> 30						
HR (95% CI) ^b^	1	1.69 (0.58–2.34)	1.51 (0.77–2.94)	1.35 (0.69–2.65)	1.37 (0.70–2.66)	0.63
**Other cancer**						
No. of deaths	81	99	110	96	124	
Age-adjusted HR (95% CI) ^a^	1	1.24 (0.92–1.66)	1.36 (1.02–1.82)	1.17 (0.87–1.57)	1.55 (1.17–2.05)	0.09
Multivariate-adjusted HR (95% CI) ^b^	1	1.23 (0.21–1.65)	1.32 (0.99–1.77)	1.11 (0.82–1.50)	1.41 (1.05–1.89)	0.08
**Age**						
< 60 years						
HR (95% CI) ^b^	1	1.05 (0.71–1.54)	1.11 (0.76–1.62)	1.07 (0.73–1.57)	1.38 (0.95–2.00)	0.10
> 60 years						
HR (95% CI) ^b^	1	1.45 (0.91–2.32)	1.58 (1.00–2.50)	1.08 (0.66–1.76)	1.25 (0.77–2.05)	0.82
**BMI**						
< 30						
HR (95% CI) ^b^	1	1.26 (0.90–1.77)	1.31 (0.94–1.83)	1.17 (0.83–1.42)	1.42 (1.02–1.99)	0.10
> 30						
HR (95% CI) ^b^	1	1.07 (0.58–1.99)	1.35 (0.74–2.46)	0.81 (0.40–1.63)	1.15 (0.59–2.23)	0.94
**Dihydrochalcones**						
**Cancer**						
No. of deaths	506		220	228	199	
Age-adjusted HR (95% CI) ^a^	1		0.68 (0.53–0.89)	0.96 (0.77–1.21)	0.83 (0.65–1.05)	0.24
Multivariate-adjusted HR (95% CI) ^b^	1		0.88 (0.75–1.03)	0.89 (0.76–1.04)	0.84 (0.71–0.99)	0.03
**Age**						
< 60 years						
HR (95% CI) ^b^	1		0.78 (0.62–0.97)	0.99 (0.80–1.21)	0.80 (0.64–1.01)	0.95
> 60 years						
HR (95% CI) ^b^	1		1.01 (0.80–1.28)	0.81 (0.63–1.04)	0.94 (0.74–1.21)	0.31
**BMI**						
< 30						
HR (95% CI) ^b^	1		0.84 (0.71–1.00)	0.87 (0.73–1.04)	0.84 (0.69–1.01)	0.04
> 30						
HR (95% CI) ^b^	1		1.04 (0.70–1.53)	0.97 (0.66–1.42)	0.75 (0.51–1.12)	0.20
**GI cancer**						
No. of deaths	278		143	117	105	
Age-adjusted HR (95% CI) ^a^	1		0.89 (0.76–1.04)	0.89 (0.76–1.05)	0.79 (0.67–0.93)	0.006
Multivariate-adjusted HR (95% CI) ^b^	1		1.05 (0.86–1.29)	0.84 (0.67–1.04)	0.85 (0.67–1.07)	0.07
**Age**						
< 60 years						
HR (95% CI) ^b^	1		0.91 (0.68–1.21)	0.82 (0.60–1.10)	0.84 (0.61–1.16)	0.17
> 60 years						
HR (95% CI) ^b^	1		1.20 (0.90–1.60)	0.90 (0.65–1.23)	0.88 (0.63–1.23)	0.26
**BMI**						
< 30						
HR (95% CI) ^b^	1		1.04 (0.83–1.29)	0.79 (0.62–1.01)	0.85 (0.66–1.09)	0.07
> 30						
HR (95% CI) ^b^	1		1.08 (0.62–1.87)	1.09 (0.64–1.85)	0.73 (0.41–1.29)	0.40
**Other cancer**						
No. of deaths	228		77	111	94	
Age-adjusted HR (95% CI) ^a^	1		1.05 (0.86–1.29)	0.84 (0.67–1.04)	0.76 (0.61–0.95)	0.009
Multivariate-adjusted HR (95% CI) ^b^	1		0.67 (0.52–0.87)	0.96 (0.76–1.20)	0.83 (0.65–1.07)	0.26
**Age**						
< 60 years						
HR (95% CI) ^b^	1		0.63 (0.45–0.89)	1.18 (0.89–1.56)	0.77 (0.55–1.08)	0.58
> 60 years						
HR (95% CI) ^b^	1		0.75 (0.50–1.12)	0.68 (0.45–1.03)	1.03 (0.71–1.49)	0.63
**BMI**						
<30						
HR (95% CI) ^b^	1		0.60 (0.44–0.81)	0.98 (0.76–1.26)	0.83 (0.63–1.09)	0.35
>30						
HR (95% CI) ^b^	1		1.01 (0.59–1.73)	0.85 (0.49–1.48)	0.80 (0.46–1.38)	0.37

The most important food sources of flavonoid subclasses in the northeast of Iran are represented in Table 5. As can be seen, tea, vegetables, and fruits are the main sources for most of the flavonoid subclasses.

**Table 5:** Main Dietary Sources of Each Flavonoid Subclass

Flavonoid Subclasses	Three Main Dietary Sources	% Contribution to Subclass
**Flavonols**	Black tea	60
	Onion	20
	apple	7
**Flavan-3-ols**	Black tea	88
	Chocolate	0.7
	Apple	0.5
**Flavanones**	Orange	96
	Lemon	0.5
	Tomato	0.2
**Flavones**	Potato	30
	Cucumber	13
	lettuce	10
**Isoflavonoids**	Soybean	97
	Beans	0.4
	Peanuts	0.2
**Anthocyanidins**	Pomegranate	30
	Cherry	15
	Grape	11

## Discussion

In this large-scale prospective cohort study, an inverse association was observed between dietary intakes of flavanones, flavones, isoflavonoids, and dihydrochalcones and risk of all-cause mortality. In contrast, total flavonoid intake and three other subclasses of flavonoids did not show a noteworthy protective effect with all-cause mortality. Furthermore, the participants with a high intake of flavanones and flavones had a lower risk of CVD mortality compared to those with lower intake. Regarding cancer mortality, isoflavonoids and dihydrochalcones were the only groups that conferred a protective effect.

The mean total flavonoid intake in this cohort study was 640 mg/d. Comparing with other populations in the US and some European countries (with total flavonoid intake of 300–400 mg/d) [20–22], the people of the northeast of Iran have a higher intake of flavonoids which is due to their higher consumption rate of tea and plant-based foods. However, the flavonoids intake of some other populations, like Australians, is even higher (about 800 mg/d) [11]. Like many other regions, tea, vegetables, and fruits were the main food sources of flavonoids in the northeast of Iran; however, since consumption of some fruits like berries or alcoholic drinks like wine is limited in this region, unlike some other studies [23], these items are not considered as an important source of flavonoids.

In general, the investigators are unanimous in maintaining that flavonoids or flavonoid-rich foods have protective effects regarding all-cause mortality or chronic disease [24], but there are also some important controversies over the details. In the present study, after adjusting for confounding variables, no significant association was seen between total flavonoid intake and all-cause mortality, CVD mortality, or cancer mortality. In consonance with our findings, investigators in Nurses’ Health Study II [12] and Iowa Women’s Health Study [25], have also reported a null association between flavonoid intakes and mortality risk. On the other hand, several other studies have shown a strong correlation between increased total-flavonoid intake and reduced risk of all-cause mortality [11, 20, 26]. The incongruent result of these studies could be justified if the complexity of different methods of flavonoids intake estimation and the variations between dietary patterns and flavonoids intakes of different populations are taken into account. Moreover, bioavailability of flavonoids may differ between individuals because of the differences between their gut microbiota composition, dietary habits and etc. [27].

Regarding flavonoid subclasses, we found an inverse association between flavanones, flavones, isoflavonoids, and dihydrochalcones intakes and the risk of all-cause mortality. Based on our findings a higher intake of these flavonoids can minimize the risk of all-cause mortality by 10–20%. In the Moli-Sani study, the investigators reported an inverse association between higher quintiles of intake of flavones, flavanones, isoflavones, and all-cause mortality risk in women. As for men, flavonols and isoflavones intake were also inversely associated with all-cause mortality [28]. In another population-based study by Ponzo et.al., after multiple adjustments of confounding variables, being in the third tertile of flavan-3-ols, anthocyanidins, and flavanones was inversely associated with all-cause mortality [21]. In a Spanish cohort by Zamora-Ros and his colleagues, the authors demonstrated an inverse association between flavanone and flavonol intakes (but no other subleases of flavonoids) and all-cause mortality [22]. In the Blue Mountains Eye Study, the protective effect of flavan-3-ols, anthocyanidins, and proanthocyanidins against all-cause mortality was observed [11].

On the subject of the probable association between flavonoid subclass intake and CVD mortality, controversies similar to the aforementioned case exist. Different studies have reported the protective effects of different flavonoid classes, however, there is a general agreement that flavonoids can protect against cardiovascular events or CVD mortality [13]. Thus, considering all these points, it appears that introducing one or two classes of flavonoids as the more important ones and dismissing others as the less important classes in relation to all-cause mortality or CVD mortality, is not feasible. Good food sources of flavonoids are tea, citrus fruits, berries, red wine, apples, soy, legumes, and fruits and vegetables in general. These food sources contain more than one group of flavonoids and the protective effects of these foods against CVD or all-cause mortality, which are reported by numerous studies [29], are at least in part due to their flavonoid contents. That being said, distinguishing the impact of each flavonoid group on all-cause or CVD mortality risks seems to be difficult in population-based studies.

Several possible mechanisms have been proposed for cardioprotective effects of flavonoids, including antioxidant, vasodilatory, antithrombotic, anti-inflammatory, and endothelial protective roles [30]. However, because of the diversity of flavonoid subclasses in terms of physicochemical properties (lipophilicity, polarity, etc.), bioavailability and bioactivity (such as antioxidant capacity or binding at receptor sites) [31], several flavonoid subclasses may have different cardioprotective effects.

With respect to cancer prevention and cancer mortality, the drawn conclusions are different; meaning that most of the observational studies have not reported any significant association between cancer and flavonoid intake [32, 33]. In a meta-analysis of 23 studies in this area, Bo et al. did not find any viable association between dietary flavonoid intake and esophageal or colorectal cancers [34]. In another review study, Romagnolo and Selmin concluded that higher intakes of dietary flavonoids cannot result in a substantial reduction of human cancer risk [35].

In the present study, comparing the highest versus the lowest quintiles of intakes, isoflavonoids and dihydrochalcones were the only subclasses of flavonoids that showed an inverse association with the risk of cancer mortality. In a meta-analysis of 14 observational studies, women with the highest intake of soy isoflavones had a significant reduction in the risk of breast cancer as against women with the lowest intake of soy isoflavones [36]. Another meta-analysis study has also reported an inverse correlation between low prostate cancer risk and high consumption of soy products [37]. The protective effect of isoflavonoids which is observed in this study is presumably because of their phytoestrogenic properties which may interfere with the synthesis and activity of endogenous hormones, influencing hormone-dependent signaling pathways and protecting against breast and prostate cancer [38].

The strengths of this study are the prospective design, the large sample size, a relatively long-term follow-up, and the conduct of the study in a developing country with a special range of flavonoid consumption. We also provided detailed information on important risk factors and confounders that were absent from some of the earlier studies. Additionally, we used the most comprehensive polyphenol database currently available (Phenol-explorer database), which allowed us to estimate all the flavonoid subclasses. However, several limitations in the current study should be concerned. The FFQ employed in this study was not originally designed to measure flavonoid intake, thus the use of FFQ as an intake measurement method imposed some limitations on our study. Additionally, due to the fact that the participants’ information was collected at baseline only, the succeeding variations in intakes and life-course changes in dietary habits which might influence the strength of the findings, could not be tracked. Finally, flavonoids content and bioavailability of different foods depend on numerous factors such as crop variety, location, type of cultivation, maturation, processing, and storage; therefore, the generalization of western polyphenol databases for the Iranians diet can be questionable.

## Conclusion

Certain types of flavonoids such as flavanones, flavones, and isoflavonoids may decrease the risk of all-cause mortality and mortality due to CVD and cancer. However, owing to the fact that most of the flavonoid-rich foods contain a combination of different flavonoid subclasses, giving preference to just one or two groups of flavonoids would be a flawed interpretation.

## Data Availability

The data are available per reasonable request on GEMSHARE website.
